# Comparative proteomic analysis of plasma membrane proteins between human osteosarcoma and normal osteoblastic cell lines

**DOI:** 10.1186/1471-2407-10-206

**Published:** 2010-05-14

**Authors:** Zhiyu Zhang, Lijun Zhang, Yingqi Hua, Xiaofang Jia, Jian Li, Shuo Hu, Xia Peng, Pengyuan Yang, Mengxiong Sun, Fang Ma, Zhengdong Cai

**Affiliations:** 1 Musculoskeletal Oncology Center, Shanghai 10th People's Hospital, Tongji University School of Medicine, Shanghai 200072, China; 2 Shanghai Public Health Clinical Center, Shanghai 201508, China; 3 Department of Orthopaedics, The 4th Affiliated Hospital, China Medical University, Shenyang, 110032, China; 4 Changhai Hospital, Second Military Medical University, Shanghai 200433, China; 5 Department of Chemistry, Fudan University, Shanghai 200433, China

## Abstract

**Background:**

Osteosarcoma (OS) is the most common primary malignant tumor of bone in children and adolescents. However, the knowledge in diagnostic modalities has progressed less. To identify new biomarkers for the early diagnosis of OS as well as for potential novel therapeutic candidates, we performed a sub-cellular comparative proteomic research.

**Methods:**

An osteosarcoma cell line (MG-63) and human osteoblastic cells (hFOB1.19) were used as our comparative model. Plasma membrane (PM) was obtained by aqueous two-phase partition. Proteins were analyzed through iTRAQ-based quantitative differential LC/MS/MS. The location and function of differential proteins were analyzed through GO database. Protein-protein interaction was examined through String software. One of differentially expressed proteins was verified by immunohistochemistry.

**Results:**

342 non-redundant proteins were identified, 68 of which were differentially expressed with 1.5-fold difference, with 25 up-regulated and 43 down-regulated. Among those differential proteins, 69% ware plasma membrane, which are related to the biological processes of binding, cell structure, signal transduction, cell adhesion, etc., and interaction with each other. One protein--CD151 located in net nodes was verified to be over-expressed in osteosarcoma tissue by immunohistochemistry.

**Conclusion:**

It is the first time to use plasma membrane proteomics for studying the OS membrane proteins according to our knowledge. We generated preliminary but comprehensive data about membrane protein of osteosarcoma. Among these, CD151 was further validated in patient samples, and this small molecule membrane might be a new target for OS research. The plasma membrane proteins identified in this study may provide new insight into osteosarcoma biology and potential diagnostic and therapeutic biomarkers.

## Background

Osteosarcoma is the third most common cancer in childhood and adolescents and the most common primary malignancy of bone. With combination treatment (neo-adjuvant chemotherapy, surgery, and adjuvant chemotherapy), the 5-year survival for patients who do not have metastatic disease at diagnosis is 60% to 70% [1,2]. However, for patients with metastatic disease at diagnosis or with tumors showing a poor response to chemotherapy, the prognosis is still unsatisfactory (5-year disease-specific survival rates, 20%-40%), even with dose-intensive or high-dose chemotherapy [3]. Thus, it is of great importance to develop new targeted therapeutic strategies based on OS-specific proteins and find more biomarkers for diagnosis as well as prognosis prediction of this lethal disease.

At present, comparative proteomics provide a powerful approach in screening for alterations in protein levels and post-translational modifications that are associated with tumors and has culminated in the identification of many potential new therapeutic targets and an abundance of cancer-related biomarkers. However, global proteomic profiling of human OS developed very late and slowly. To our knowledge, only a few papers have reported comparative proteome research in OS, including our previous data obtain by comparative proteomic analysis of patient sera [4-8]. In some of these researches, tissue and cell lines were used. But due to the complexity and difference of proteome, low copy proteins and membrane proteins were usually undetected in whole cell or tissue. Recently, many proteomic investigations have focused on subcellular compartments [9,10]. The plasma membrane (PM) is an organized system serving as a structural and communication interface for exchanges of information and substances with the extracellular environment. The proteins on the PM act as 'doorbells' and 'doorways' playing crucial roles in cell function including intercellular communication, cellular development, cell migration, and drug resistance [6,11-13]. So it is important to systematically study the PM proteins involved in OS.

PM proteomic research of OS faces three challenges: 1) excluding the individual difference; 2) obtaining adequate and purified PM for proteomic analysis; 3) identifying low abundant proteins. In this study, MG-63 (an OS cell line) and hFOB1.19 (a SV40-immortalized normal osteoblastic cell line) were used as a comparative model for studying the proteins related to OS. PM was separated by aqueous two-phase partition. Proteins were analyzed by iTRAQ-based LC-MS/MS-based proteomics to exclude the protein bias in two-dimensional electrophoresis (2DE) [14,15]. 342 proteins were identified, out of which, 69 proteins were found to be differentially expressed for more than 1.5-fold. The expression of CD 151 antigen was further evaluated by immunohistochemistry in clinical samples. It's the first time the PM proteomics of OS was studied and CD151 antigen was found to be over-expressed in cell lines and confirmed its overproduction in OS clinical tissue, which was also observed to be up-regulated in breast cancer [16,17] but down-regulated in colorectal cancer [18]. Our results showed that sub-cellular proteomics is a useful method for selecting OS biomarkers and CD151 might be potential target for diagnosis and treatment of OS.

## Methods

### Cell culture

Human osteosarcoma cell lines MG-63, and the normal osteoblastic cell line hFOB1.19 (expressing SV40 large T antigen) were originally obtained from the American Type Culture Collection (Manassas, VA, USA). The cell lines were cultured as previously described [8] with some modification. Briefly, the cell lines were cultured in Dulbecco's Modified Eagle Medium (DMEM) (Invitrogen) with 10% FBS. For human osteoblastic cell line hFOB1.19, a 1:1 Ham's F12 medium was added to DMEM without phenol red and with 2.5 mmol/L *L*-glutamine and 0.3 mg/mL G418. All cultures were maintained in 10 cm diameter dishes in a humidified atmosphere of 5% CO_2 _at 37°C. MG-63 cells were subcultured every 2 to 3 days, and hFOB1.19 cells were subcultured every 4 to 5 days. Furthermore, the same Ham's F12 medium and DMEM with 10% FBS without G418 was used to incubate the two cell lines for the least 24 hours before harvesting them for membrane extraction to exclude any unexpected affect that the difference between culture medium will cause. About 10^8 ^cells were collected and used for PM separation.

### Patient tissue samples

All patient tissue and clinical information was collected with patients' consent after permitted by Ethics Committee of Tongji University. Eleven archival sections of formalin-fixed, paraffin-embedded primary osteosarcoma and their respective adjacent non-tumorous tissue were collected.

### Preparation of plasma membrane

The PM was isolated as previously described [19,20]. All steps were carried out at 4°C. Briefly, adherent cells (10^8^) were washed three times with PBS, scraped using a plastic cell lifter, and broken in 1 mL solution containing 0.2 mM EDTA, 1 mM NaHCO_3 _using a glass homogenizer. The nuclear and unbroken cells were removed through 200 g, the supernatant was collected, and centrifuged for 30 min at 25000 rpm. The cell pellets were resuspended in 1 mM NaHCO_3 _in an approximate ratio of 1 ml per 5 × 10^8 ^cells and used for PM separation by two-phase systems [20]. 2 g of suspended cell pellets was added to the top of 14 g of the dextran-poly(ethylene glycol) mixture (6.6% Dextran T500, 6.6% PEG 3350,0.2 M K_3_PO_4_, pH7.2). After mixed for 40 times, the tube was centrifuged for 5 min at 750 g. The PM-enriched upper phase was collected and purified again as before. The upper phase was diluted 5-fold with 1 mM sodium bicarbonate, and centrifuged at 100 000 g for 2 h in a SW32 rotor. The pellets were collected and used for purification check and proteomics.

### Protein Digest, iTRAQ Labeling, and Strong Cation Exchange Fractionation

iTRAQ labeling was done according to the kit protocol (Applied Biosystems Inc., Foster City, CA) and the previously reported by Jonghwa Jin [21,22]. Protein (100 μg) from the PM of MG-3 and hFOB1.19 cell lines was acetone precipitated overnight at -20°C and resuspended in 30 μL iTRAQ™ Dissolution Buffer (ABI, Foster City, USA). After reduction and alkylation, proteins solutions were digested overnight at 37°C with sequence grade modified trypsin (Promega) (1:10). The peptides were pooled, desalted with Sep-Pak Cartridge (Waters) and fractionated by Strong Cation Exchange (SCX) chromatography on an Ultimate HPLC system (LC Packings) using a Column(5 μm, 300 Å, 0.5 × 23 mm, Waters). Peptides were eluted with a linear gradient of 0-500 mM KCl (25% v/v acetonitrile, 10 mM KH_2_PO_4_, pH 2.8) for 60 min at a flow rate of 200 μl/min. 15 fractions were collected. The iTRAQ experiments were carried out twice: the first experiment compared MG-63 cells (115 reporter ions) and hFOB1.19 (116 reporters), while the second experiment was performed using MG-63 (115 reporters), hFOB1.19 (114 reporters).

### LC-MS Analysis

Each SCX fraction was dried down, dissolved in 0.1% formic acid, and analyzed on Qstar Pulsar™ (Applied Biosystems-MDS Sciex). Peptides were separated on a reverse-phase column packed with ZORBAX 300SB-C18 enrichment column (5 μm, 300 Å, 0.5 × 23 mm, Waters) and separated by a 75-μm-internal diameter PepMap RP column from LC Packings packed with 3-μm C18 beads with 100-Å pores. Buffer A: 5%ACN, 95% water, 0.1% FA and Buffer B: 95%ACN, 5% water, 0.1%FA. The flow rate used for separation on the RP column was 400 nl/min with gradient 5%-45% during 90 min. MS data was acquired automatically using Analyst QS 1.0 software Service Pack 8 (ABI/MDS SCIEX, Concord, Canada). An analysis survey scans were acquired from 400-1800 with up to 6 precursors selected for MS/MS from m/z 100-2000. The two most intense peaks over 30 counts, with a charge state 2-4 were selected for fragmentation. Curtain gas was set at 10, nitrogen was used as the collision gas, and the ionization tip voltage was 4000 V.

### Data analysis

Ratios of the 114.1, 115.1 and 116.1 amu signature mass tags generated upon MS/MS fragmentation from the iTRAQ™-labeled tryptic peptides were calculated using Protein Pilot (ABI, USA) (version 2.0.1) (ABI) in Analyst. The MS and MS/MS tolerances were set to 0.2 Da. The IPI databases was used for searching iTRAQ™-identified peptides. Methyl methanethiosulphonate modification of cysteines was used as a fixed modification, and one missed tryptic cleavage was allowed. All proteins identified must have ≥95% confidence and the protein confidence threshold cutoff was set to 1.3 (unused) with at least more than one peptide above the 95% confidence level. The true value for the average ratio was expressed as an error factor (EF = 10^(95% confidence interval)^) and calculated according to the reports [23,24]. An EF <2 was set for the quantification quality to be satisfied. In addition, a p-value < 0.05 was significant for protein quantification. To designate significant changes in protein expression, fold-changes >1.5 or <0.66 were set as cutoff values. Furthermore, in order to decrease the artificial error, results were "auto" bias corrected using IT115: IT114 of 1.3564. The peptide and proteins were exported, and saved as excel files.

### Bioinformatics

The theoretical isoelectric point (pI) and molecular weight (MW) and Grand average of hydropathicity (GRAVY) were calculated through inhouse developed software. The sub-cellular location and function of the identified proteins were elucidated by UniProt knowledgebase (Swiss-Prot/TrEMBL) and Gene Ontology Database. The mapping of putative transmembrane helices (TMHs) in the identified proteins was carried out using the transmembrane hidden Markov model (TMHMM) algorithm, available at http://www.cbs.dtu.dk/services/TMHMM. A protein-protein interaction network was done by STRING software through inputting IPI number http://string.embl.de.

### Immunohistochemistry

Immunohistochemistry (IHC) was carried as previously described [25,26]. The sections were then dehydrated by passage through a series of ethanol and embedded in paraffin, and incubated with anti-CD151 (Abcam, clone 11G5a Cambridge, MA, USA) in a humidified chamber overnight and washed in PBS for 3 times followed by incubated with secondary antibody 30 min and detected using a liquid 3, 3'-diaminobenzidine (DAB) staining kit from Gene Tech Company. Sections were counterstained with Hematoxylin-exon, dehydrated, mounted in Permount (Fisher Scientific) and four representative fields were captured digitally by light microscopy using an Olympus BX40 equipped with a logenE PAS9000. The antigen density was counted by Image-Pro Plus v6.2 software (Media Cybernetics, Inc., Bethesda, MD). The density of all positive staining in each photograph was measured and summed up then normalized with total cell number counted to give an expression rate of antigen.

### Statistical Analysis

Protein expression levels of immunohistochemistry were compared between groups with two-way T-test.

## Results

### Production and characterization of PM derived from cell lines

**Figure 1:**
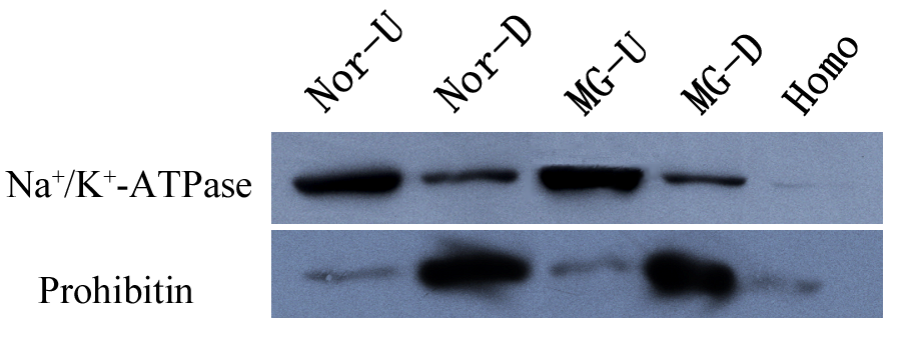
**Verification of PM through Western Blot**. Fifty micrograms of homogenate, upper and down-phase proteins were separated in 11.5% SDS-PAGE and transferred to a PVDF membrane. The blots were probed with antibodies against organelle-specific proteins: anti-Na^+^/K^+^-ATPase for PM; anti-prohibitin for mitochondria. Homo, U and D mean homogenate, upper-phase and down-phase respectively. "Nor" stands for hFOB1.19 and "MG" stand for MG-63. Na^+^/K^+^-ATPase enriched upper-phase was collected as PM.

Although the growth characteristic of the two cell lines--MG-63 and hFOB1.19 is different, the cells were cultured with similar nutrition. In order to decrease the difference caused by culture nutrition, the cells were cultured for at least 24 hours in the same nutrition before they were collected. About 10^8 ^cells were used for PM separation. In this work, a combination of differential centrifugation and aqueous two-phase partitioning [20] was used to separate plasma membrane. Two phases were obtained after centrifugation, including upper and down-phase. Fractions containing PM were determined based on the enrichment of a PM marker enzyme--Na^+^/K^+ ^ATPase and the decrease of a mitochondrial marker-Prohibitin. As shown in Figure 1, PM was enriched in the upper phase by comparing the signal strength of upper phase (Nor U and MG U) with that of down phase (Nor D and MG D). According to the results analyzed by Image J software http://rsb.info.nih.gov/ij, PM was enriched for 11.2 or 15.3-fold in upper phase and 6.5 or 3.9-fold in down-phase in hFOB1.19 or MG-63 cell line compared with homogenate. While mitochondrial was increased for 1.2 or 1.4-fold in upper phase and 6.9- or 6.3-fold in the down phase in hFOB1.19 or MG-63 cell line. Basically, the purification of PM was successful in both cell lines.

### Identification of differentially expressed proteins

Since 2D-LC-MS/MS method provides a powerful alternative to gels especially in detecting hydrophobic proteins, a newly developed iTRAQ technique was used here to compare protein expression between MG-63 and hFOB 1.19 cells. After duplicated LC-MS/MS analysis, 342 proteins were quantified, which had a p-values > 95% confidence level (ProtScore > 1.3) and at least more than one peptide above the 95% confidence level. 60 of them were detected in both independent experiments, (Additional file 1). 66% (226 of 342) proteins were identified by more than 5 peptides, 10% (36 out of 342) by 4 peptides; 11% (38 out of 342) by 3 peptides, 8% (27 out of 342) by 2 peptides and only 5%(17 out of 342)by one peptide (Additional file 2).

**Figure 2:**
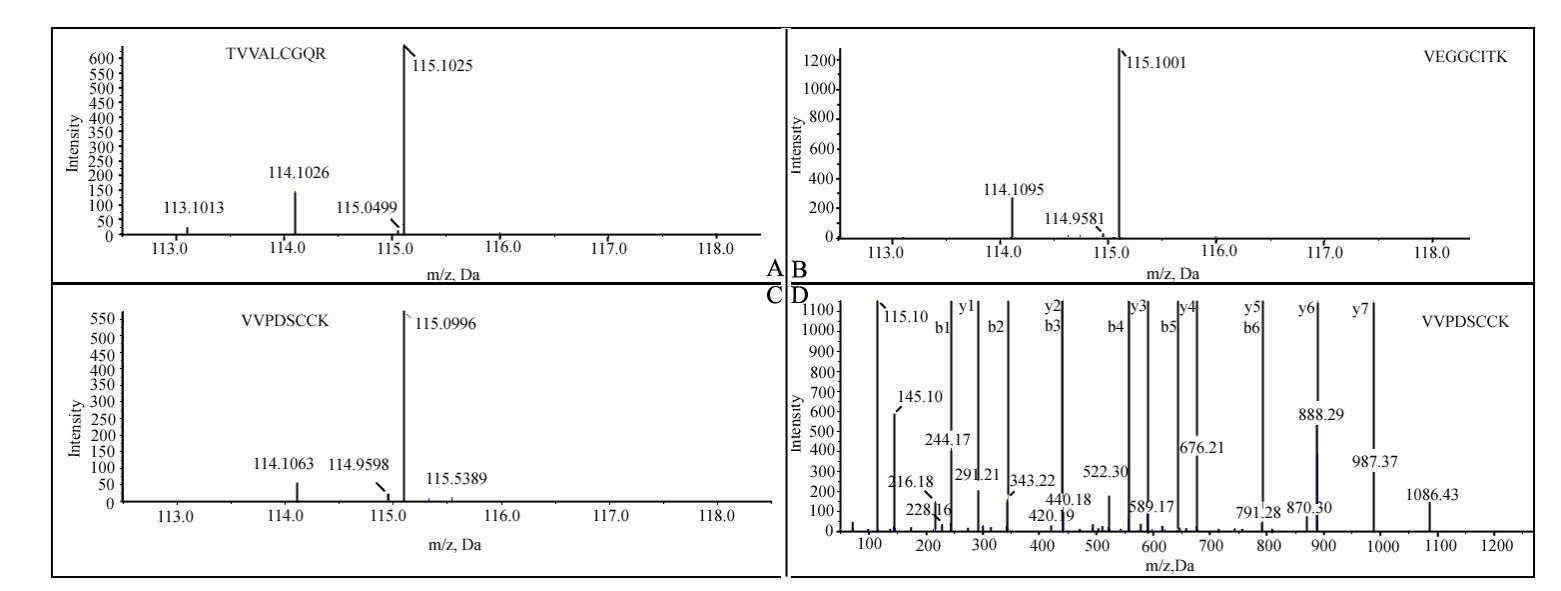
**MS and MS/MS spectra of CD151**. Examples of MS and MS/MS spectra of a 3.67-fold up-regulated protein--CD151 antigen, illustrating the degree of relative quantitative measurement is consistency. MG-63 and hFOB 1.19 were labeled with 115- and 114- reporter isobaric reagent respectively. Three quantified tryptic peptides (from a total of 5 non-redundant peptides) were shown in figure A, B and C. A, The MS spectra of a peptide-TVVALCGQR with m/z of 568.81 (z = 2); B, The MS spectra of a peptide VEGGCITK, m/z = 570.80 and z = 2; C, The MS spectra of a peptide VVPDSCCK, m/z = 615.78 and z = 2. D, The MS/MS spectra of the peptide (VVPDSCCK), related b ion and y ion were shown.

The following criteria were adopted: (1) cutoff iTRAQ ratios of fold-change for protein expression were >1.5 for up-regulation and <0.66 for down-regulation; (2) A protein had to be quantified with at least three spectra (allowing generation of a p-value), a p-value < 0.05; (3) An EF <2 was set for the quantification quality to be satisfied. According to these criteria, a total of 68 differentially expressed proteins were screened from the two experiments. In the first experiment, 30 proteins were found to be changed for more than 1.5-fold, including 10 proteins up-regulated and 20 down-regulated with a p-value < 0.05. In the second, 48 proteins (P < 0.05) were changed, 21 proteins up-regulated and 27 down-regulated. Combination of two independent experiments showed 10 identified proteins including 6 up-regulated proteins and 4 down-regulated (as shown in Additional file 3). Representative MS/MS spectra for three peptides identified from CD151 antigen are shown in Figure 2. Consistent changes were found in the three peptides (Figure 2A, B and 2C). Almost total y or b ions were detected in the sequence (Figure 2D).

### Bioinformatic analysis of differentially expressed proteins

#### Physicochemical characteristics of the identified Proteins

36% of these differential proteins are transmembrane proteins, including 3 proteins with more than 10 transmembrane helices (TMHs),4 protein with 4 TMHs, 3 proteins with 2 TMHs, and 12 proteins with 1 TMH (Additional file 3). Of the 79 differentially expressed proteins, 13.0% hydrophobic proteins (with positive HP values up to 0.77) were identified (Additional file 3). According to the annotations from UniProt knowledgebase (Swiss-Prot/TrEMBL) and Gene Ontology Database, 68.1% (47 of 69) differential proteins located in plasma membrane including proteins annotated as membrane, single-pass type I membrane protein anchored to membrane, intermediate filament, actin cytoskeleton, microtubule, cell-cell adherent junction as well as plasma membrane. The other proteins locate in other sub-cellular organelles such as nucleus (14.5%), cytoplasm (1.4%) and endoplasmic reticulum (ER)-Golgi intermediate (1.4%), or have unknown location (10 proteins) (Additional file 3, Figure 3A). Furthermore, we simply analyzed the protein function annotated by GO database and found that the following biological processes were more frequently changed: proteins with binding activity (including protein binding, ATP binding, DNA binding, etc) (81.69%), cell structure (18.31%), and signal transduction (18.31%) (Additional file 4, Figure 3B).

**Figure 3:**
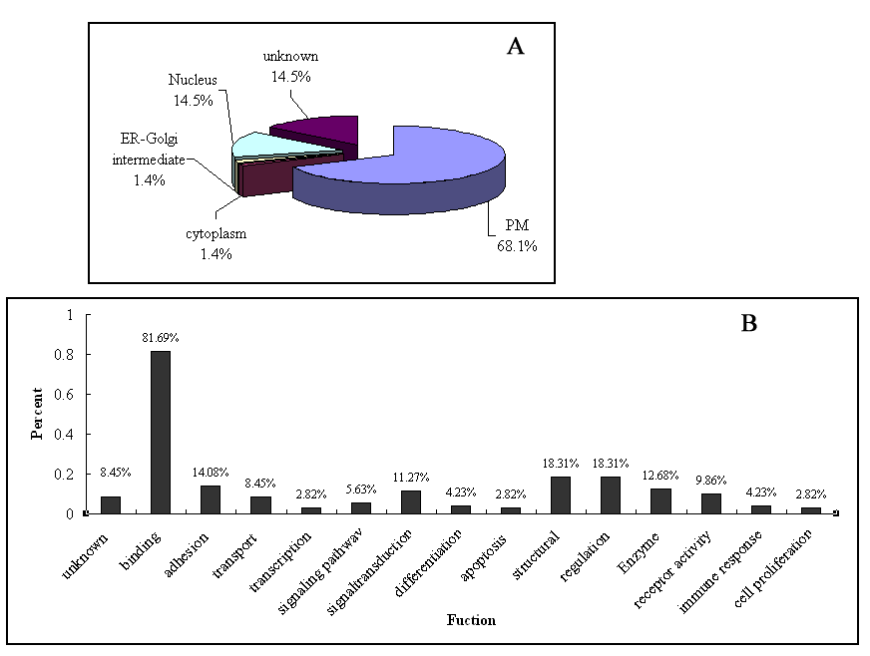
**Bioinformatic analysis of differentially expressed proteins**. A. The sub-cellular location distribution, B. Function distribution graph of identified proteins. GO database were used for the data analysis.

#### Protein-protein interaction analysis

**Figure 4:**
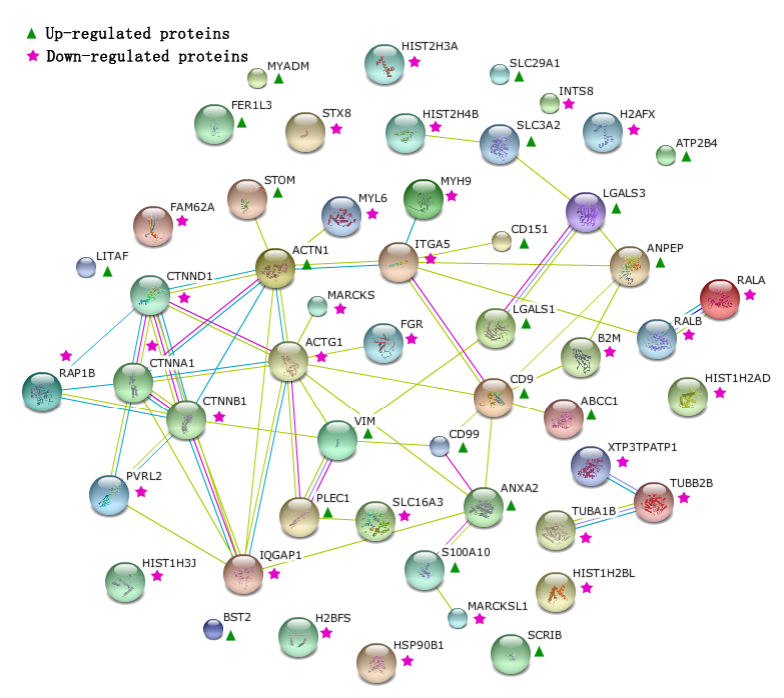
**The protein-protein interaction network analyzed by String software**.

Carcinogenesis is a very complex process and mediated by a lot of proteins. In this work, we want to know how PM proteins interact with each other and how they effect cell's function in OS. To investigate these issues, we searched the string database for protein interaction, against proteins we identified as seeds. As shown in Figure 4, 52 seed proteins were involved protein-protein interaction. Of which 21 were up-regulated proteins marked with "Δ", 31 were down-regulated highlighted by "★". In the network, some proteins with consistent changes interacts with each other, such as the down-regulated proteins (RAP1B-CTNNA1-PVRL2-IQGAP1 -CTNNB1-CTNND1-ACTG1-FGR), the up-regulated proteins (PLEC1-VIM-CD99- CD9-ANPEP-LGALS3-LGALS1). However, in the all network, up-regulated proteins usually interact with the down-regulated to constitute a big network, for example, CD151 (Δ)-ITGA5 (★)-ACTN1 (Δ)-CTNND1 (★). These seed proteins have important function in signal transduction, cell adhesion, etc. For example, CD151 can activate the PI3K pathway and promote neovascularization via the PI3K pathway [27]. CTNNB1 (Catenin beta-1, down-regulated in OS) is involved in the regulation of cell adhesion and in signal transduction through the Wnt pathway [28]. CTNND1 may regulate the cell adhesion properties of both C and E-cadherin, and implicate both in cell transformation by SRC and in ligand-induced receptor signaling through the EGF [29].

According to the above bioinformatics analysis, CD151 antigen, was selected for further study due to 1) its PM location; 2) hydrophobic character (HP of 0.33; TMHs of 4); 3) important function (having protein binding activity, taking part in PI3K pathway and involving in protein-protein interaction (CD151, up-regulated; ITGA5, down-regulated).

### Immunohistochemistry

**Figure 5:**
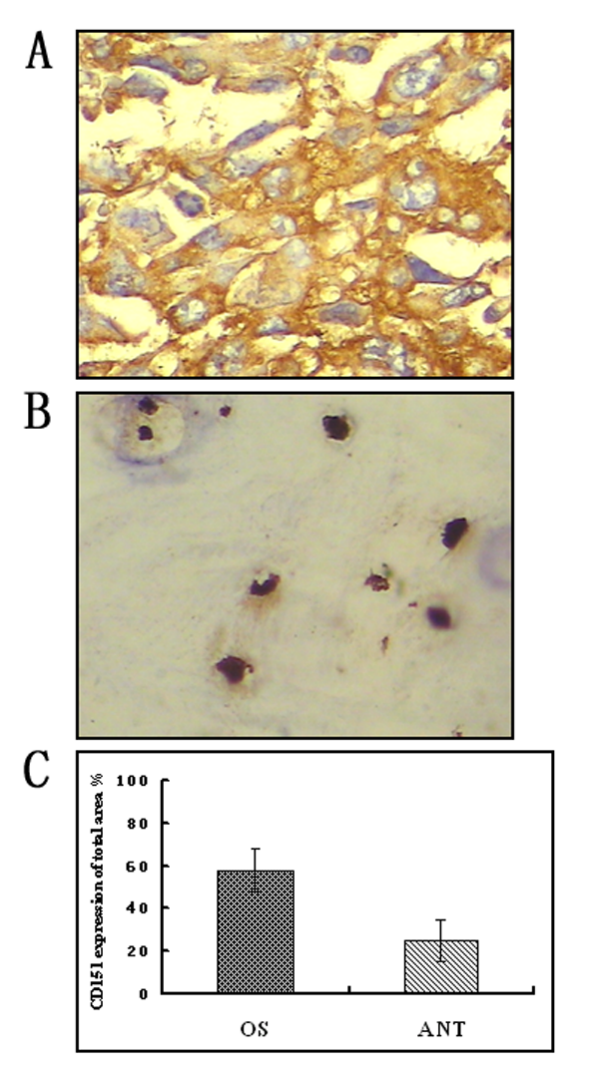
**Immunohistochemistry analyzing the expression of CD151**. A. OS samples, B. Adjacent non-tumorous tissues. Immunoreactivity of CD151 was located in the plasma membrane. C. Semi-quantification analysis of OS samples and adjacent non-tumorous tissues. Integrated optical density (IOD) are 57.9 ± 16.9% and 25.1 ± 13.1% respectively (P = 0.0002). IOD of all positive staining in each photograph was measured by its ratio to total area.

Our differentially expressed proteins were detected in established cell lines. To further verify the results obtained from cell lines, an immunohistochemical study was carried on OS tissue samples and their non-tumorous adjacent tissue using anti-CD151 antibody. The relative positive signal in OS (Figure 5A) is much more than that in the controls (Figure 5B). The semi-quantification analysis showed that the expression of CD151 in OS and its control is 57.9 ± 16.9% (n = 11) and 25.1 ± 13.1% (n = 11) respectively (Additional file 5, Figure 5C), which revealed a good correlation between CD151 antibody staining and the proteome expression profiles. Significant difference between these OS and its controls (p = 0.0002) was detected, with 2-fold up-regulated in OS according to semi-quantitative analysis. This result validated our finding from proteomic probing of differential plasma protein. CD151 may be a key molecule in regulating the tumorigenesis and migration of osteosarcoma due to its membrane protein function.

## Discussion

Proteomics holds great promise in contributing to the prevention and cure of cancer because it provides unique tools for high-throughput screening of biomarkers and therapeutic targets. As such, proteomics can help to translate basic science discoveries into the clinical practice of personalized medicine [30,31]. However, its application in osteosarcoma is very limited [4-8,32-36] due to the difficulty in sample collection and protein extraction directly from bone tissue. So far, there are only several proteomic researches using osteosarcoma cell lines or serum sample of OS patients. No sub-cellular proteomic research of OS was found so far. Due to the complexity of proteome in whole tissue or cells, many low abundant proteins will be undetected, for example membrane proteins. Thus, in this study, we presented an OS plasma membrane proteomic research combining differential proteins identified in cell lines with differential protein verification in tumor sample.

In order to identify more membrane proteins, iTRAQ™--stable isotope labeling, in conjunction with tandem MS was used. iTRAQ is a high content proteomic technique for substrate degradomics, can be used to label 4 or 8 samples simultaneously, and has been widely used in proteomic research [37-39]. In our study, through comparing the plasma membrane proteome of OS cell (MG-63) and human osteoblastic cell (hFOB1.19), 342 proteins were identified, including well known plasma membrane markers such as sodium/potassium-transporting ATPase subunit beta-3, 5'-nucleotidase precursor, Flotillin-1, Voltage-dependent anion-selective channel protein 1 as well as several CD antigen-CD151, CD55, CD81, CD63, etc. In this work, 68 differentially expressed proteins were identified, of which, 69.8% located in plasma membranes, including CD151, CD99, etc. Consistent with previous PM studies [22,40,41], we also identified non-PM proteins in our PM because of the biochemical isolation of PM and maybe the multiple locations of proteins. Further, due to the use of iTRAQ combining LC-MS, 31.9% of differentially expressed proteins have more than one TMHs.

According to the annotation from GO database, the differential proteins identified in this experiment function as binding, signal transduction, immune response, and angiogenesis, etc, and most might be involved in the factors related to cancer progress. The proteins with binding function were mostly detected, and cell structure and signal transduction proteins were second most. We also detected several differential proteins involved immune response as well as proteins related to angiogenesis. These results indicated that following conclusion. First, it is multi-factor progress for osteosarcoma development, and many proteins are involved in. Second, proteins with binding, cell structure, and signal transduction activities are affected greatly in carcinogenesis. Finally, sub-cellular proteomic research can be used to find proteins related to cancer.

Through bio-information analysis such as protein location, function and protein-protein interaction, one protein--CD151 was selected for further verification. Immunohistochemistry staining validated the up-regulation of CD151 in OS. As annotated by UniProtKB/Swiss-Prot registered as P48509, CD151 antigen, a multi-pass membrane protein, interacts with integrins alpha3beta1, alpha5beta1, alpha3beta1 and alpha6beta4, with CD9 and CXCR1(CD181). CD151 functions as an important regulator of communication between tumor cells and endothelial cells, and might be as a potentially novel prognostic marker and target for therapy in breast cancer [16], biomarkers for assessment of malignancy in gingival squamous cell carcinoma (GSCC) [42], renal cell carcinoma [43] and metastasized colorectal carcinoma [18]. However, no research about CD151 in OS was reported and this was the first report about its over-expression in osteosarcoma cell line and sample, and can enlarge the knowledge of CD151 in cancer. CD151 might be a potentially novel biomarker and therapeutic target for OS. Further functional experiments are worth doing.

## Conclusions

To summary, in this work, we reported a sub-cellular proteomic research combining PM purification, iTRAQ label, LC-MS separation and identification in OS cell lines with clinical verification in patient's tissues and plasma. Many PM proteins with several TMs were detected to be differentially expressed. CD151 were identified and verified to be a candidate of biomarker and therapeutic target for OS. Our research might provide some clue to understand the mechanism of OS progress and offer novel biomarkers for OS diagnosis and treatment.

## Abbreviations

OS: osteosarcoma; iTRAQ: isobaric tag for relative and absolute quantitation; LC-MS: Liquid chromatography-mass spectrometry; HCT: High Capacity Trap; HE: Hematoxylin and Eosin.

## Competing interests

The authors declare that they have no competing interests.

## Authors' contributions

ZZ, YH, and ZC designed the study and experiment protocols. LZ directed proteomic research. LZ, ZZ, YH, and ZC wrote the first draft of the manuscript together. MS and JL participated in IHC staining and analysis. XJ and XP finished the separation of PM, protein identification and bioinformatics analysis. PY participated in protein identification. FM and SH finished WB experiments. ZZ, YH participated in bioinformatics analysis. All authors have read and approved the final manuscript.

## Pre-publication history

The pre-publication history for this paper can be accessed here:

http://www.biomedcentral.com/1471-2407/10/206/prepub

## Supplementary Material

Additional file 1 **Table S1-The list of all the proteins identified in this work**. Two separated iTRAQ label plus mass spectrometry identification and quantification experiments were carried out in our study. Proteins identified twice are highlighted in bold italic letters. Click here for file

Additional file 2 **Table S2-The list of all the peptides detected by mass spectrometry**. Only proteins with at least one peptide with confidence more than 95% were considered significant. Click here for file

Additional file 3 **Table S3-The differentially expressed proteins identified in this work**. Click here for file

Additional file 4 **Table S4-The differentially expressed proteins with protein functions**. Click here for file

Additional file 5 **Table S5 Statistic results of immunohistochemistry of osteosarcoma tissue samples and non-tumours control tissues**. Click here for file
