# Supplementary material for: Comparative proteomic analysis of plasma membrane proteins between human osteosarcoma and normal osteoblastic cell lines
Source: BMC Cancer. 2010 May 14;10:206. doi: 10.1186/1471-2407-10-206 (PMC2880991; doi:10.1186/1471-2407-10-206)
Supplement: Additional file 3 — Table S3-The differentially expressed proteins identified in this work. [file 1471-2407-10-206-S3.DOC]

**Table S3- The differentially expressed proteins identified in this work**

| Number^a^ | Total | %Cov(95)^b^ | Accession | Ratio^c^ (OS/Nor.) | PVal^d^ | EF^e^ | experiment | Abbreviation in interaction^f^ | PI | HP | Mw | TMHs | Location |
| --- | --- | --- | --- | --- | --- | --- | --- | --- | --- | --- | --- | --- | --- |
| 153 | 6.46 | 5.59 | IPI00006666.1 | 0.62 | 4.00E-02 | 1.17 | second | SLC16A3 | 8.22 | 0.66 | 49469 | 12 | PM |
| 236 | 1.56 | 2.97 | IPI00009225.1 | 0.62 | 2.61E-02 | 1.17 | second | STX8 | 4.91 | -0.471 | 26906.7 | 1 | PM |
| ***221** | **3.41** | **0.84** | **IPI00009342.1** | **0.65** | **0.00E+00** | **1.17** | **second** | **IQGAP1** | **6.08** | **-0.49** | **189251** | **0** | **PM** |
| 240 | 18.55 | 2.9 | IPI00014898.2 | 4.12 | 0.00E+00 | 1.32 | first | PLEC1 | 5.73 | -0.66 | 531738 | 0 | PM |
| 55 | 3.4 | 13.59 | IPI00015148.3 | 0.54 | 1.00E-02 | 1.34 | first | RAP1B | 5.64 | -0.39 | 20825 | 0 | PM |
| 306 | 2.01 | 1.51 | IPI00016871.1 | 0.41 | 1.00E-02 | 1.75 | second | FGR | 5.4 | -0.36 | 59479 | 0 | unknown |
| 137 | 4.43 | 6.27 | IPI00017292.1 | 0.51 | 3.00E-02 | 1.72 | first | CTNNB1 | 5.52 | -0.18 | 85497 | 0 | PM |
| 48 | 4.12 | 14.29 | IPI00018534.4 | 0.45 | 0.00E+00 | 1.19 | second | HIST1H2BL | 10.31 | -0.7 | 13952 | 0 | nucleus |
| *****219*** | ***17.46*** | ***2.3*** | ***IPI00019502.3*** | ***0.63*** | ***0.00E+00*** | ***1.22*** | ***second*** | ***MYH9*** | ***5.49*** | ***-0.85*** | ***226530*** | ***0*** | ***PM*** |
| *****219*** | ***8*** | ***3.42*** | ***IPI00019502.3*** | ***0.64*** | ***2.00E-02*** | ***1.39*** | ***first*** | ***MYH9*** | ***5.49*** | ***-0.85*** | ***226530*** | ***0*** | ***PM*** |
| 11 | 13.08 | 28.8 | IPI00021440.1 | 0.64 | 0.00E+00 | 1.12 | first | ACTG1 | 5.3 | -0.2 | 41793 | 0 | PM |
| 103 | 2 | 8.33 | IPI00026241.1 | 5.66 | 1.73E-02 | 1.82 | first | BST2 | 5.43 | 0.029 | 19768.91 | 1 | PM |
| 242 | 4.27 | 2.86 | IPI00027230.3 | 0.36 | 3.16E-02 | 1.9 | second | HSP90B1 | 4.76 | -0.713 | 92468.8 | 0 | cytosol |
| 72 | 6 | 11.01 | IPI00031370.3 | 0.49 | 4.00E-02 | 1.95 | first | TUBB2B | 4.78 | -0.41 | 49953 | 0 | PM |
| 166 | 2 | 5.3 | IPI00101961.2 | 0.46 | 1.00E-02 | 1.43 | second | - | 4.47 | -0.38 | 17119 | 0 | unknown |
| 225 | 2.25 | 3.11 | IPI00102685.1 | 1.92 | 0.0202 | 1.58 | second | MYADM | 8.53 | 0.77 | 35273.5 | 8 | PM |
| 16 | 2.15 | 23.53 | IPI00171611.7 | 0.43 | 0.00E+00 | 1.08 | first | HIST2H3A | 11.26 | -0.6 | 15388 | 0 | nucleus |
| *****7*** | ***3.41*** | ***10.31*** | ***IPI00183695.9*** | ***1.66*** | ***0*** | ***1.08*** | ***second*** | ***S100A10*** | ***6.82*** | ***-0.416*** | ***11187*** | ***1*** | ***unknown*** |
| *****7*** | ***4.02*** | ***35.05*** | ***IPI00183695.9*** | ***2.08*** | ***3.32E-02*** | ***1.86*** | ***first*** | ***S100A10*** | ***6.82*** | ***-0.36*** | ***11203.1*** | ***1*** | ***unknown*** |
| 110 | 6 | 7.84 | IPI00215948.4 | 0.56 | 0.00E+00 | 1.11 | first | CTNNA1 | 5.94 | -0.37 | 100071 | 0 | PM |
| 308 | 2 | 1.46 | IPI00215980.1 | 0.44 | 0.00E+00 | 1.5 | second | PVRL2 | 5 | -0.16 | 51359 | 1 | PM |
| 248 | 5.74 | 2.68 | IPI00217170.1 | 1.84 | 1.04E-05 | 1.24 | second | ATP2B4 | 6.19 | -0.114 | 137920.1 | 8 | PM |
| 165 | 3.83 | 5.34 | IPI00217519.3 | 0.49 | 0.00E+00 | 1.1 | second | RALA | 6.65 | -0.62 | 23567 | 0 | PM |
| 26 | 7.44 | 19.58 | IPI00219037.5 | 0.42 | 0.00E+00 | 1.29 | second | H2AFX | 10.73 | -0.36 | 15145 | 0 | nucleus |
| *9 | 4.73 | 5.92593 | IPI00219219.3 | 1.6 | 1.74E-04 | 1.24 | second | LGALS1 | 5.33 | -0.151 | 14715.7 | 0 | PM |
| 111 | 3.53 | 7.83 | IPI00219301.7 | 0.26 | 0.00E+00 | 1.4 | second | MARCKS | 4.47 | -0.71 | 31555 | 0 | PM |
| *****19*** | ***2.1*** | ***2.7777778*** | ***IPI00219682.6*** | ***1.89*** | ***3.57E-07*** | ***1.2*** | ***second*** | ***STOM*** | ***10.31*** | ***-0.7*** | ***13952*** | ***0*** | ***nucleus*** |
| *****19*** | ***6*** | ***21.53*** | ***IPI00219682.6*** | ***3.73*** | ***0*** | ***1.74*** | ***first*** | ***STOM*** | ***10.31*** | ***-0.7*** | ***13952*** | ***0*** | ***nucleus*** |
| 63 | 12 | 11.92 | IPI00219869.1 | 0.58 | 0.00E+00 | 1.11 | first | CTNND1 | 5.8 | -0.71 | 105894 | 0 | PM |
| *****29*** | ***21.95*** | ***18.82*** | ***IPI00221224.6*** | 4.31 | ***0.00E+00*** | ***1.24*** | ***first*** | ***ANPEP*** | 5.3 | -0.32 | 109540 | 1 | PM |
| *****29*** | ***25.72*** | ***11.17*** | ***IPI00221224.6*** | 6.55 | ***0.00E+00*** | ***1.14*** | ***second*** | ***ANPEP*** | 5.3 | -0.32 | 109540 | 1 | PM |
| 5 | 5.4 | 42.31 | IPI00255316.5 | 0.47 | 2.00E-02 | 1.62 | first | HIST1H2AD | 10.9 | -0.47 | 14107 | 0 | nucleus |
| 85 | 6.08 | 9.88 | IPI00298851.4 | 3.67 | 0.00E+00 | 1.39 | second | CD151 | 7.44 | 0.33 | 28295 | 4 | PM; |
| 197 | 4.61 | 2.67 | IPI00306604.5 | 0.45 | 0.00E+00 | 1.25 | second | ITGA5 | 5.5 | -0.22 | 114536 | 1 | PM |
| 335 | 8.49 | 0.61579063 | IPI00398779.4 | 1.88 | 1.80E-02 | 1.66 | second | PLEC1 | 5.74 | -0.665 | 531790.7 | 0 | PM |
| 274 | 3.24 | 2.07792204 | IPI00413696.5 | 1.51 | 1.77E-03 | 1.19 | second | - | 6.82 | 0.599 | 35213.6 | 6 | PM |
| 341 | 2 | 0.41 | IPI00413958.4 | 0.62 | 2.00E-02 | 1.29 | first | - | 5.68 | -0.31 | 287216 | 0 | PM |
| *****18*** | ***11.7*** | ***14.59*** | ***IPI00418471.6*** | ***3.24*** | ***0.00E+00*** | ***1.23*** | ***first*** | ***VIM*** | ***5.06*** | ***-0.82*** | ***53652*** | ***0*** | ***PM*** |
| *****18*** | ***22.62*** | ***22.1*** | ***IPI00418471.6*** | ***3.46*** | ***0.00E+00*** | ***1.23*** | ***second*** | ***VIM*** | ***5.06*** | ***-0.82*** | ***53652*** | ***0*** | ***PM*** |
| 284 | 4 | 1.87424421 | IPI00425566.1 | 1.88 | 6.09E-03 | 1.34 | first | SCRIB | 5.01 | -0.447 | 174884.9 | 0 | PM |
| 228 | 1.4 | 3.0837005 | IPI00444204.1 | 0.6 | 6.74E-02 | 1.97 | first | RALB | 8.82 | -0.627 | 25710.3 | 0 | unknown |
| *****2*** | ***11.7*** | ***50.49*** | ***IPI00453473.6*** | ***0.44*** | ***0.00E+00*** | ***1.13*** | ***second*** | ***H1ST2H4B*** | ***11.36*** | ***-0.52*** | ***11367*** | ***0*** | ***nucleus*** |
| *****2*** | ***6*** | ***29.13*** | ***IPI00453473.6*** | ***0.44*** | ***0.00E+00*** | ***1.15*** | ***first*** | ***HIST2H4B*** | ***11.36*** | ***-0.52*** | ***11367*** | ***0*** | ***nucleus*** |
| ***3** | **29.07** | **39.2330378** | **IPI00455315.4** | **1.62** | **6.41E-18** | **1.09** | **first** | **ANXA2** | **7.57** | **-0.524** | **38604.0** | **0** | **PM** |
| 83 | 4.69 | 10.29 | IPI00465070.7 | 0.54 | 0.00E+00 | 1.12 | second | HIST1H3J | 11.12 | -0.58 | 15404 | 0 | nucleus |
| 244 | 1.64 | 2.80000009 | IPI00465431.7 | 1.6 | 9.14E-03 | 1.22 | second | LGALS3 | 8.58 | -0.395 | 26152.3 | 0 | nucleus |
| 301 | 4.74 | 1.61 | IPI00473136.3 | 0.45 | 1.00E-02 | 1.61 | second | CTNNA1 | 5.98 | -0.36 | 102635 | 0 | PM |
| 27 | 4 | 19.05 | IPI00477495.3 | 0.41 | 1.00E-02 | 1.41 | first | H2BFS | 10.36 | -0.75 | 13944 | 0 | nucleus |
| 322 | 4.03 | 0.99 | IPI00478565.2 | 0.51 | 0.00E+00 | 1.44 | second | - | 6.62 | -0.39 | 112261 | 2 | PM |
| 181 | 4 | 4.49 | IPI00550382.2 | 4.45 | 0.00E+00 | 1.64 | first | SLC29A1 | 8.48 | 0.39 | 58963 | 11 | PM |
| *****35*** | ***17.5*** | ***15.873*** | ***IPI00604710.2*** | ***1.81*** | ***1.09E-12*** | ***1.13*** | ***second*** | ***SLC3A2*** | ***4.89*** | ***-0.147*** | ***67994*** | ***1*** | ***PM*** |
| *****35*** | ***18.52*** | ***16.507937*** | ***IPI00604710.2*** | ***1.99*** | ***1.04E-05*** | ***1.25*** | ***first*** | ***SLC3A2*** | ***5.46*** | ***-0.143*** | ***61613.9*** | ***1*** | ***unknown*** |
| 216 | 2 | 3.59 | IPI00641181.5 | 0.24 | 0.00E+00 | 1.66 | second | MARCKSL1 | 4.68 | -1.16 | 19529 | 0 | PM |
| 105 | 2.04 | 8.28 | IPI00642948.1 | 2.73 | 0.00E+00 | 1.39 | second | CD99 | 4.38 | -0.31 | 17128 | 2 | PM |
| 186 | 2 | 4.34782617 | IPI00645256.1 | 1.66 | 1.58E-04 | 1.2 | second | LITAF | 5.99 | 0.015 | 17106.7 | 0 | PM |
| *****139*** | ***32.1*** | ***6.20117*** | ***IPI00645867.2*** | ***1.74*** | ***3.23E-20*** | ***1.09*** | ***second*** | ***FER1L3*** | ***5.84*** | ***-0.456*** | ***234708.7*** | ***1*** | ***PM*** |
| *****139*** | ***19.58*** | ***5.2734375*** | ***IPI00645867.2*** | ***1.83*** | ***2.20E-09*** | ***1.12*** | ***first*** | ***FER1L3*** | ***5.87*** | ***-0.457*** | ***233476.5*** | ***1*** | ***PM*** |
| 247 | 2.46 | 2.69 | IPI00647896.1 | 0.45 | 0.00E+00 | 1.4 | second | XTP3TPATP1 | 4.8 | -0.34 | 41742 | 0 | PM |
| 238 | 4 | 2.92 | IPI00719178.1 | 4.98 | 0.00E+00 | 1.49 | first | ABCC1 | 6.39 | 0.14 | 172609 | 16 | PM |
| *****255*** | ***1.79*** | ***0.63*** | ***IPI00746655.1*** | ***0.49*** | ***1.00E-02*** | ***1.43*** | ***second*** | ***FAM62A*** | ***5.53*** | ***-0.27*** | ***124003*** | ***2*** | ***PM*** |
| *****255*** | ***4*** | ***2.6*** | ***IPI00746655.1*** | ***0.57*** | ***1.00E-02*** | ***1.24*** | ***first*** | ***FAM62A*** | ***5.53*** | ***-0.27*** | ***124003*** | ***2*** | ***PM*** |
| 133 | 9.89 | 6.56455159 | IPI00759776.1 | 1.54 | 2.10E-03 | 1.28 | first | ACTN1 | 5.01 | -0.55 | 35411.9 | 0 | unknown |
| 25 | 15.4 | 19.71 | IPI00792677.1 | 0.43 | 0.00E+00 | 1.12 | second | TUBA1B | 4.95 | -0.24 | 46313 | 0 | PM |
| ***30** | **2.62** | **5.26** | **IPI00795937.1** | **3.93** | **0.00E+00** | **1.37** | **second** | **CD9** | **5.32** | **0.27** | **14764** | **1** | **unknown** |
| 70 | 2 | 11.0344827 | IPI00796366.1 | 0.5 | 4.90E-02 | 1.98 | first | MYL6 | 5.01 | -0.704 | 26706.9 | 0 | unknown |
| 31 | 2.26 | 8.4 | IPI00796379.1 | 0.63 | 0.00E+00 | 1.18 | second | B2M | 6.05 | -0.35 | 13697 | 0 | PM |
| 330 | 2.03 | 0.82 | IPI00797345.1 | 0.66 | 2.00E-02 | 1.36 | second | INTS8 | 6.35 | -0.14 | 111116 | 0 | nucleus |
| ***124*** | ***5.73*** | ***4.34782617*** | ***IPI00827658.1*** | 1.7 | ***0.00027997*** | ***1.24*** | ***first*** | - | 5.13 | -0.770 | 81553.5 | 1 | PM |
| 280 | 8.39 | 1.94 | IPI00867588.1 | 0.58 | 0.00E+00 | 1.18 | second | - | 5.39 | -0.5 | 249302 | 0 | ER-Golgi intermediate |
| 251 | 6.25 | 0.97 | IPI00871932.1 | 0.64 | 0.00E+00 | 1.17 | second | - | 5.37 | -0.77 | 276138 | 0 | PM |
| 102 | 11.23 | 8.36 | IPI00872684.1 | 0.34 | 0.00E+00 | 1.31 | second | - | 5.87 | -0.97 | 69372 | 0 | unknown |
| *****125*** | ***11.99*** | ***7.12*** | ***IPI00872814.1*** | ***0.52*** | ***0.00E+00*** | ***1.17*** | ***second*** | ***-*** | ***6.08*** | ***-0.97*** | ***67687*** | ***0*** | ***PM*** |
| *****125*** | ***5.28*** | ***4.511*** | ***IPI00872814.1*** | ***0.61*** | ***2.92E-02*** | ***1.33*** | ***first*** | ***-*** | ***6.09*** | ***-0.972*** | ***67686.8*** | ***0*** | ***PM*** |
| 4 | 7.99 | 43.65 | IPI00873444.1 | 0.65 | 0.00E+00 | 1.09 | second | - | 7.3 | -0.47 | 78952 | 0 | PM |
| 321 | 7.89 | 1.01 | IPI00879810.1 | 0.66 | 0.00E+00 | 1.24 | second | - | 5.23 | -0.78 | 284948 | 0 | PM |
| 39 | 12.71 | 15.09 | IPI00894325.1 | 0.07 | 0.00E+00 | 1.9 | second | - | 6.53 | -0.508 | 41318 | 1 | PM |
| 8 | 23.64 | 33.07 | IPI00894498.1 | 0.59 | 0.00E+00 | 1.05 | second | - | 5.93 | -0.549 | 13539 | 0 | PM |
| 28 | 17.26 | 18.83 | IPI00895803.1 | 4.43 | 0.00E+00 | 1.79 | second | - | 6.09 | -0.533 | 41373 | 1 | PM |

a, The proteins list in Number column are identified in this work. Proteins identified in both of the two independent experiments and met the differential standard were highlighted with “**” and bold italic characters. Proteins identified in both experiments but met the differential standard only in one of them were highlighted with “*” and bold characters.

b, The protein coverage with 95% confidence.

c, The ratio of identified protein in OS cell to osteoblast cell according to the reporters of 115/116 or 115/114. The bias were auto corrected through IT115/ IT116 or IT 115/ IT 114

d, The P value of 115/116 or 115/114

e, EF means error factor (95% confidence).

f, “-” means the proteins not detected in string interaction software.

g, Abbreviation: PM: plasma membrane
